# Deconstructing virus condensation

**DOI:** 10.1371/journal.ppat.1009926

**Published:** 2021-10-14

**Authors:** Nora Lopez, Gabriela Camporeale, Mariano Salgueiro, Silvia Susana Borkosky, Araceli Visentín, Ramon Peralta-Martinez, María Eugenia Loureiro, Gonzalo de Prat-Gay

**Affiliations:** 1 Centro de Virología Animal (CEVAN), Consejo Nacional de Investigaciones Científicas y Tecnológicas (CONICET)–Universidad Abierta Interamericana, Buenos Aires, Argentina; 2 Fundación Instituto Leloir, IIB-BA Conicet, Buenos Aires, Argentina; University of Pittsburgh, UNITED STATES

## Abstract

Viruses have evolved precise mechanisms for using the cellular physiological pathways for their perpetuation. These virus-driven biochemical events must be separated in space and time from those of the host cell. In recent years, granular structures, known for over a century for rabies virus, were shown to host viral gene function and were named using terms such as viroplasms, replication sites, inclusion bodies, or viral factories (VFs). More recently, these VFs were shown to be liquid-like, sharing properties with membrane-less organelles driven by liquid–liquid phase separation (LLPS) in a process widely referred to as biomolecular condensation. Some of the best described examples of these structures come from negative stranded RNA viruses, where micrometer size VFs are formed toward the end of the infectious cycle. We here discuss some basic principles of LLPS in connection with several examples of VFs and propose a view, which integrates viral replication mechanisms with the biochemistry underlying liquid-like organelles. In this view, viral protein and RNA components gradually accumulate up to a critical point during infection where phase separation is triggered. This yields an increase in transcription that leads in turn to increased translation and a consequent growth of initially formed condensates. According to chemical principles behind phase separation, an increase in the concentration of components increases the size of the condensate. A positive feedback cycle would thus generate in which crucial components, in particular nucleoproteins and viral polymerases, reach their highest levels required for genome replication. Progress in understanding viral biomolecular condensation leads to exploration of novel therapeutics. Furthermore, it provides insights into the fundamentals of phase separation in the regulation of cellular gene function given that virus replication and transcription, in particular those requiring host polymerases, are governed by the same biochemical principles.

## Introduction

Eukaryotic viruses are intracellular parasites in constant coevolution with their host cells. With small genomes and few protein products, they rely on the cellular machinery to execute their life cycle consisting of a series of steps starting with viral entry, and followed by postentry events that lead to the synthesis of viral proteins and genomes, and finally to virion assembly and release, according to particular strategies. These events are orchestrated with exquisite precision, balancing the need to hijack cellular chemical machinery while keeping the cell healthy enough to complete their infectious cycle. These tasks include blocking the innate immune response, preventing apoptosis and generating an adequate environment for genome transcription and replication, for which a physical separation away from the host cytosolic (or nuclear) components is required. Such compartmentalization must be finely regulated to coordinate transcription and replication with nucleocapsid and particle assembly.

To fulfill genome replication, viruses evolved diverse genomic frameworks. In many RNA viruses, such as negative-stranded viruses (NSVs), replication is accomplished by virus-encoded enzymes packaged into the mature viral particle, and which are readily available for initiating viral RNA synthesis upon infection. In those, viral proteins are translated from subgenomic viral mRNAs. In contrast, some positive-sense RNA viruses that do not transcribe subgenomic RNAs translate their viral proteins, including replication enzymes, directly from the messenger-sense genome. Irrespective of specific strategies employed by each viral group to take control of the cell machinery, fundamental questions arise on how de novo synthesized viral proteins and nucleic acids are organized to direct virus multiplication and assembly. Will these diffuse around the cytosol or nuclei? How is genome replication spatiotemporally organized? What mechanisms govern the physical partitioning of gene function and the formation and packaging of nucleocapsids into the virion?

A vast number of complex biochemical processes in the cell can organize into dynamic and finely tuned structures known as membrane-less organelles (MLOs). An increasingly large number of these are reported to be formed by liquid–liquid phase separation (LLPS) of macromolecules, giving place to biomolecular condensates (BMCs) that partake in a wide range of physiological and pathological processes across life kingdoms [1–3]. Examples of BMCs are stress granules (SGs), nucleoli, P granules, Cajal bodies, among others extensively reviewed [4–8].

At the time MLOs were being described as liquid-like structures, viral replication was also found to take place within granular structures with dynamic properties evoking LLPS. The scope of this review is to show that this appears to be a widespread phenomenon in virus life cycles, describe some examples, and provide a general biochemical background for LLPS and biomolecular condensation in connection with viral perpetuation. In addition, we discuss how these mechanisms operate for viral gene function and other aspects of virus biology that benefit from these dynamic structures.

## Replication sites: Different names, similar entities

A common feature observed during the course of infection by many viruses, whether they replicate in the cytoplasm or nucleus of the infected cells, is the formation of electrondense structures. These structures have been referred to as viral factories (VFs), viral inclusions, inclusion bodies (IBs), replication organelles, viral replication compartments (VRCs), transcription–replication complexes, virosomes, or viroplasms. One of the first characterization of VFs was provided for Poxviruses, enveloped DNA viruses that replicate exclusively in the cytoplasm [9]. Poxvirus factories are sites where protein synthesis, viral mRNA transcription, and DNA replication take place and are gradually enwrapped by rough endoplasmic reticulum (ER) membranes, later dispersed as viral assembly starts [10].

Nuclear-replicating double-stranded (ds) DNA viruses share common strategies including the formation of VRCs that drive a profound remodeling of subnuclear compartments, such as the promyelocitic leukemia nuclear bodies, Cajal bodies, and nucleoli [11–13]. For example, during cytomegalovirus infection, VRCs coalesce to form a single compartment that may occupy most of the nuclear space [13]. These viruses can also interact with DNA damage response (DDR) machinery, frequently hijacking DNA repair and replication factors for their own benefit [14].

Factories formed by Rotavirus, a member of the Reoviridae family that is associated with viral gastroenteritis in young children and infants worldwide, have been extensively characterized. Transcription and replication of the viral segmented dsRNA genome, as well as packaging of the newly synthesized pregenomic RNA, take place into membrane-less cytosolic electron-dense inclusions termed viroplasms. These complex structures are composed of genomic dsRNAs packed together with viral and cellular proteins, including ER chaperones [15], lipid droplets-associated proteins [16], and ribonuclear proteins [17], and are organized in concentric layers [18].

NSVs are enveloped and contain either one (nonsegmented) or several single-stranded RNA segments. The viral genome is enwrapped by multiple copies of the nucleoprotein (N) forming viral ribonucleoprotein (vRNP) complexes, which associate with the viral RNA–dependent RNA polymerase (vRdRp). Almost all NSV replicate in the cytoplasm of the infected cells, where they form VFs typically involving at least N protein and the viral polymerase and, in some cases, additional viral proteins, such as, for example, the phosphoprotein of nonsegmented NSV (nsNSV), which is an essential cofactor of the polymerase [19]. They have also been shown to colocalize with a number of host proteins including those involved in cellular mRNA metabolism, ribosomal subunit proteins, SG proteins, translation initiation factors, and key factors of the interferon pathway among others [20–23].

### Replication sites confined to membranous structures

A hallmark of positive-sense RNA viruses is the association of viral replication with an extensive rearrangement of cellular membranes including formation of invaginated vesicles or spherules, or double-membrane vesicles (DMVs). For example, within the Flaviviridae family, dengue virus and Zika virus build up a scaffold of single-membrane invaginated vesicles in the ER, which are connected to the cytosol by small pores [24,25]. They are assumed to be the site of genome replication, as they contain dsRNA replication intermediates and viral replicase complex proteins [26]. Hepatitis C virus, in turn, creates a membranous web of DMVs, which protrude from the ER and incorporate viral RNA and replicase proteins [24].

DMV-type VFs have also been described for the distantly related Picornaviridae, Arteriviridae, and Coronaviridae families [27–29]. Recent analyses of severe acute respiratory syndrome coronavirus-2 (SARS-CoV-2)-infected cells have revealed that DMVs, which contain RNA, are connected to the cytosol through crown-shaped pores, which would provide a transport route for newly synthesized viral genomes and messenger RNAs into the cytoplasm [30,31]. Altogether, the extensive ER rearrangement organized by many positive single-stranded RNA viruses to facilitate viral replication is not limited to animal viruses, but also applies to members of the Bromoviridae and Tombusviridae families of plant viruses that generate vesicle-like membrane invaginations (spherules) where cellular proteins are recruited and replication takes place [32,33].

A number of positive-stranded RNA viruses, particularly SARS-CoV2 [34] and Flaviviruses, including hepatitis C virus, dengue virus, Zika virus, West Nile virus, and Japanese encephalitis virus [35], have been reported to co-opt lipid droplets as an auxiliary platform for viral replication. Viral recruitment of lipid droplets, which are dynamic cytoplasmic organelles containing neutral lipids and proteins, has been proposed to assist the RNA polymerase activity [36] as well as to sustain scaffolding for genome encapsidation process [37].

Overall, viral proteins synthetized in the cytosol must come together to form viral replication sites, which require physical and functional separation from host cell processes. What is their exact nature, and how is their assembly and disassembly regulated?

## Biomolecular condensation and liquid–liquid phase separation

### Principles of LLPS

**Fig 1:**
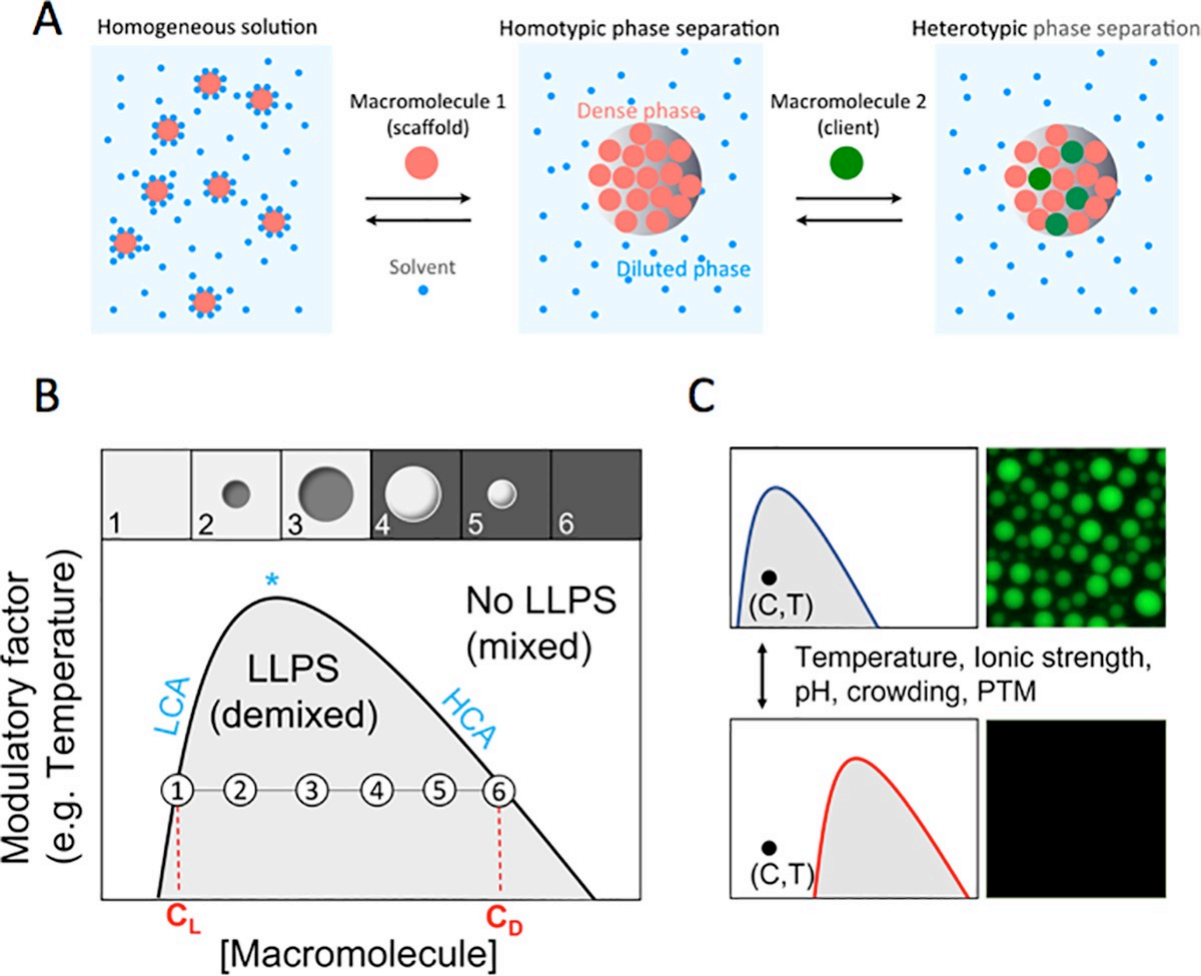
Overview of LLPS, phase diagram, and modulation. **(A)** Under certain conditions, a solvated macromolecule (scaffold) undergo homotypic LLPS and concentrate in a distinct liquid compartment (dense phase). One or more additional macromolecules (clients) can partition into the new phase through heterotypic LLPS. **(B)** A phase diagram describes the phase behavior of a binary (macromolecule and solvent) or multicomponent system (at least 2 macromolecules) as a function of macromolecular concentration or any other physicochemical factor that may modulate its condensation tendency. Here, we present a concentration vs temperature phase diagram for a binary system. A phase boundary (black curve, known as binodal) defines whether the system is in a 1-phase regime (mixed solution) or in a 2-phase regime (demixed solution). All coordinate pairs of concentration and temperature that lie beneath the phase boundary (gray) give rise to LLPS. The phase boundary maximum is the critical point (star), above which a homogeneous solution is seen at any macromolecular concentration. The critical point divides the phase boundary in 2 segments known as low concentration arm (LCA, left) and high concentration arm (HCA, right). The LCA defines the concentration of the diluted or light phase (CL), whereas the HCA defines the concentration of the dense phase (CD). Increasing total concentration in the light phase above the concentration threshold only changes the relative volumes between phases (i.e., droplets become larger at the expense of the diluted phase; see Fig 3). The top panel of (B) illustrates this phenomenon: (1) macromolecule concentration at threshold, no LLPS. At higher concentration, small droplets form (2) growing in size (3) as concentration increases. Eventually, the volume of the dense phase is higher than the diluted phase, so surface tension dictates the formation of diluted droplets surrounded by dense phase (4). After this inversion boundary, increasing concentration decreases the diluted droplets size (5) until a 1-phase regime of dense solution only is achieved (6). **(C)** Modulatory effectors such as PTMs or pH operate by altering the forces that drive droplet formation, thus changing the phase boundary. All of these modulatory effects may act in favor or against LLPS, depending on the nature of the interactions involved. For instance, addition of a negative charge by phosphorylation has the potential to engage components in electrostatic attractive or repulsive forces. C,T, concentration, temperature; HCA, high concentration arm; LCA, low concentration arm; LLPS, liquid–liquid phase separation; PTM, posttranslational modification.

Under certain solvent conditions, and above a determined threshold concentration, a homogenous polymer–solvent mixture can separate or “demix” into 2 phases, one enriched in the polymer at the expense of partial depletion of the same polymer from the diluted phase, leading to the formation of liquid-like droplets coexisting with the dilute surrounding. This well-known principle in synthetic polymer chemistry [38] applies to biological macromolecules, mainly proteins and nucleic acids, the key players in BMCs. In simple and general thermodynamic terms, the condensed phase is a lower entropy state (molecules have less freedom) than the homogeneous mixture or diluted phase. Thus, phase separation may only take place if the confinement-associated entropic cost is counterbalanced by favorable macromolecular interactions. This scenario is possible in poor solvent conditions, i.e., scarce polymer solubility and polymer–polymer interactions being more energetically favorable than polymer–solvent interactions. In other words, under phase separation conditions, the polymer or macromolecule has more affinity for itself (or other macromolecules) than for the solvent (Fig 1A). In the context of the highly crowded environment of the cell, corresponding to protein concentrations of ca 150 mg/mL [39], some proteins are often above their solubility. This supersaturation state is prone to rapid changes triggered by different effectors giving rise to phase separation [40].

A demixing process consisting of 1 polymer or macromolecular component and solvent is referred to as homotypic, whereas 2 or more components are referred to as heterotypic LLPS (Fig 1A). Hallmarks of LLPS are the formation of spherical liquid droplets, which increase in size upon increasing component concentration in the surrounding phase. These droplets are dynamic, they fuse and coalesce, are frequently reversible, and are studied using a broad range of methods [41,42].

### Characteristics of the macromolecules and interactions involved

Proteins involved in LLPS usually exhibit a subset of the following features: (i) intrinsic disorder; (ii) multivalence; (iii) modularity; (iv) nucleic acid binding; (v) oligomeric nature; and (vi) dynamic conformation. Viral proteomes are known to be overabundant in intrinsically disordered proteins (IDPs), or regions (IDRs), providing functional flexibility and genome economy for interfering with cellular processes in favor of their infectious cycle [43]. Within IDPs, the so-called “low complexity regions” (LCRs) stand out as highly disordered polypeptides, some of them exhibiting a strong tendency for homotypic LLPS [44]. Low complexity refers to a high relative abundance of a small subset of the 20 amino acids; consequently, no significant folding or persistent secondary structure is possible. Folding inability may be due to the low abundance of aromatic and hydrophobic amino acids making it difficult for a hydrophobic core to be formed. Multivalent, low affinity (≤0.2 kcal/mol) interactions between certain amino acids drive LLPS in LCRs. They often involve aromatic (F, Y, W), polar (Q, N, S), or charged residues (D, E, R, K) that engage in π–π stacking or π–cation [45], dipolar [46], or complementary charge-based interactions [45,47]. On the other hand, multidomain modular proteins include a variety of examples with strong tendency to LLPS. These are composed of multiple folded domains separated by disordered linkers. They often form discrete and stable oligomers, which are an important driving force for LLPS by increasing avidity and lowering the entropic cost of confinement [5].

Although cellular condensates such as the nucleolus include a diversity of components [48], many proteins can undergo homotypic LLPS spontaneously without other components [45,47,49]. These molecules are referred to as scaffolds, which are able to recruit clients, components that do not form LLPS spontaneously but are a fundamental part of the condensate [5]. A combination of ratios between scaffold and clients, high or low binding affinities and very importantly high valence, dictate the compositional control of BMCs [5].

A large number of the condensates that make up MLOs involve RNA as a central player. These include SGs, processing bodies (PBs) [50], paraspeckles [51], and the nucleolus [52], among others, grouped together as ribonucleoprotein granules [53,54]. In these, RNA binding proteins and RNAs act as essential drivers for the condensate, since its polyanion nature confers multiple binding sites, i.e., high valency. Although less studied, similar considerations apply to DNA, as an anionic multivalent polymer capable of multiple weak electrostatic interactions driving BMC, in particular those associated to transcriptional control [55]. In line with this, emerging evidence highlights the importance of an interplay between chromatin organization, chromatin binding, and LLPS in the formation of transcription factories [56].

### Modulation of BMCs assembly and disassembly

According to ideal polymer LLPS theory [57], an equilibrium exists between condensate droplets and the dilute phase. Thus, cellular droplet formation and dissolution can be achieved by varying macromolecular concentration above or below a concentration threshold (Fig 1B). The higher the concentration threshold, the lower the tendency for a macromolecule to condensate (Fig 1C). In turn, condensation tendency is governed by the sum of attractive and repulsive forces involved in macromolecular interactions. Thus, cellular control of droplet formation and dissolution can also be achieved by enabling/disabling interactions and modulating their affinities, either by covalent or noncovalent modifications on the scaffold itself (posttranslational modifications (PTMs), oligomerization state, ligands, or cosolutes) or through physicochemical changes in the milieu (temperature, ionic strength, osmolarity, pH, and crowding). All these modulatory effectors may act in favor or against LLPS depending on the nature of the interactions involved [58–60]. For instance, PTMs such as phosphorylation or methylation may affect the charge state or binding properties of a protein, thus imposing drastic effects on its phase-separating behavior [61].

### Material properties

While many condensates exhibit highly dynamic liquid-like properties, others behave as less-dynamic, noncoalescent gels or even functional solids [62]. Thus, BMCs span a spectrum of viscoelastic properties suited to fulfill diverse functions that depend on markedly different diffusional rates, such as enhancing enzymatic activities or transiently storing biomolecules. Furthermore, LLPS is frequently mediated by metastable elements that may alternatively follow irreversible aggregation routes that derive in the formation of amorphous aggregates or regular fibrils, a phenomenon known as droplet maturation, aging, or liquid-to-solid transition [63,64]. As the function of BMCs depends on their material properties, cellular maintenance of droplet fluidity relies on ATP-consuming processes that may involve chaperones, disaggregases, and RNA-helicases [65–67].

## Viral factories as dynamic liquid-like entities

Cumulative evidence suggests that assembly of VFs is often regulated by multivalent interactions that allow gradual incorporation of specific components [68]. Here, we describe some of the most representative examples of VFs that have been characterized to date as being liquid-like and potentially LLPS-driven.

### Negative-stranded RNA viruses

nsNSVs are classified within the order Mononegavirales, which includes important human and animal pathogens such as rabies virus (RABV), measles virus (MeV), and Ebola virus (EBOV) [69]. Most of these viruses may utilize an endocytic pathway to enter cells and release their ribonucleoprotein complex (RNP) into the cytosol. All nsNSVs share a common genomic structure and code for 5 to 11 proteins depending on the virus species. The vRNP consists of the viral RNA genome encapsidated by N and associated with the vRdRp (L) and the nonenzymatic phosphoprotein (P). For some nsNSVs, additional viral proteins are also associated with the vRNP, such as the processivity factor M2-1 of pneumoviruses, or the equivalent transcription enhancer VP30 and the VP24 of filoviruses. Mononegavirales N proteins share structural characteristics; they present 2 globular domains with the RNA bound in a central groove [70]. P proteins (VP35 for filoviruses) differ significantly in sequence, size, and oligomeric state. However, a modular and dynamic architecture of a tight oligomeric domain flanked by IDRs is conserved (reviewed in [71,72]).

Initial observations of cytoplasmic viral condensates formed during nsNSV infections come from RABV. The viral genome is approximately 12 kb long and encodes 5 proteins: N, P, L, a matrix protein (M), and a glycoprotein (G), gene order being 3′-N-P-M-G-L-5′ in the viral RNA [73]. First studies of rabies infections by histological analyses of infected tissues revealed cytoplasmic inclusions, called Negri bodies (NBs) after their discovery in 1903 by Adelchi Negri [74]. NBs recruit all the viral replication machinery together with M and cellular proteins such as the focal adhesion kinase FAK, the chaperone Hsp70, and the eukaryotic cytosolic chaperonines CCTα and CCTγ [75–77]. NBs were shown to be involved in transcription, replication, and viral assembly and provided the first evidence that VFs are liquid-like and could be generated by LLPS in vivo [76,78]. Indeed, NBs display characteristics similar to LLPS-driven MLOs since they are small and spherical during early times after infection, they fuse and become more heterogeneous over time, and they can be dissolved by hypotonic shock [78]. Viral nucleocapsids are ejected from these condensates, possibly due to a decrease in their solubility within NBs, and are transported further away along the microtubule network to form new VFs [78]. Alternatively, vRNPs may outflow the NBs through the formation of a double membrane that surrounds the factories in late stages of infection, allowing direct budding of virions [73].

Shortly after the evidence that NBs are liquid-like VFs, condensates displaying similar properties were described for vesicular stomatitis virus (VSV), MeV, and human metapneumovirus [79–81]. MeV condensates, as for other nsNSVs, are composed of viral RNA, N, P, and L viral proteins. In addition, they contain the viral nonstructural C protein, which is involved in counteracting the host cell immune system [82], as well as the WD repeat-containing protein 5 (WDR5) host protein [23]. Interestingly, MeV condensates evolve from liquid to gel-like structures as they mature over time during infection, suggesting that physical properties may change to fulfill the viral replication cycle [80]. Another example illustrating the relevance of LLPS in nsNSV life cycle is respiratory syncytial virus (RSV), the main cause of bronchiolitis in infants worldwide. In RSV-infected cells, condensates are formed where viral RNA and N, P, and L proteins concentrate together with the viral transcription antiterminator M_2-1_. Only found in the Pneumoviridae family, M_2-1_ interacts with P and RNA [83] and plays a key role as an elongation factor, to enable synthesis of full-length viral mRNAs [84]. Termed IBs, these condensates have been characterized as sites where replication and transcription occur [85]. Interestingly, it has been shown that M_2-1_ and viral mRNA are transiently located in dynamic subcompartments within IBs, named inclusion body–associated granules (IBAGs), which exclude N, P, and L and genomic RNA. In vitro studies demonstrated that upon disassembly of IBAGs, M_2-1_ and mRNA are released from IBs, suggesting that M_2-1_ directs viral mRNAs to the cytosol for translation [85]. IBs also recruit M to the vRNP complex during infection, possibly inhibiting viral transcription to facilitate virion assembly and packaging [86].

Overall, nsNSV factories display properties of liquid organelles and concentrate viral RNA and at least N, P, and L viral proteins [87], but how proteins interact with each other and trigger VF assembly may differ along virus families. In vitro systems revealed the minimal requirements for the formation of these condensates. In the case of RABV, MeV, and RSV, coexpression of N and P proteins in transfected cells are necessary and sufficient to form spherical inclusions [78,80,85,88,89]. In the case of VSV, L forms inclusions by itself, but all 3 L, N, and P are required to trigger cytoplasmic phase separation [79], and for EBOV, the expression of N protein is sufficient for the generation of inclusions in transfected cells [90].

In contrast to other nsNSVs, Borna disease virus (BDV), a member of the Bornaviridae family, replicates noncytopathically in the nucleus and establishes persistent infections. The membrane-less BDV factories contain viral RNA and N and P proteins and are assembled within the nucleus in close association with host chromatin [91]. Photobleaching techniques to evaluate BDV components that make up these spherical nuclear inclusions revealed that P protein is mobile and shuttles between inclusions, suggesting LLPS properties [92].

### Influenza virus

RNA viruses that replicate in the nucleus include members of the Orthomyxoviridae family, such as influenza A virus (IAV), a pathogen of worldwide impact. Following uncoating, IAV nucleocapsids are imported into the nucleus where viral mRNAs are transcribed and viral genome is replicated. Newly produced vRNPs, exported to the cytoplasm, come together and accumulate in membrane-less foci (vRNP hotspots) that can fuse together as they are transported to the plasma membrane, the site of virus assembly [93]. Cellular Rab11a-containing endosomes are thought to serve as platforms for the trafficking of vRNPs to the plasma membrane via the microtubule network [94]. These vRNP hotspots have been recently described as displaying characteristics of liquid-like organelles in terms of shape, dynamics, ability to deform, and reactivity to physiological changes and have been proposed to increase vRNA concentration at specific sites to facilitate the early stages of viral genome packaging [95].

### Retroviruses

Retroviruses positive-sense single-strand RNA genome is retrotranscribed to a DNA molecule that is integrated into the host genome. The integrated provirus serves as template for the cellular RNA polymerase II (Pol II)-directed viral RNA synthesis, and viral assembly occurs at the plasma membrane, where proteins associated with dimers of genomic RNA condense to form immature budding particles [96]. LLPS has been implicated in different events of the human immunodeficiency virus type 1 (HIV-1) cycle. Viral infection was shown to rearrange intranuclear compartments and induce clustering of viral DNA, viral RNA, and host proteins in large nuclear foci, which originate in the absence of chromosomal integration and might represent viral reservoirs or a way to escape from the innate immune response [97,98]. Likewise, in vitro and in cellulo experiments have revealed that the HIV-1 nucleocapsid protein (NC) displays the ability to drive liquid-like condensates. NC is a small, basic nucleic acid binding protein derived from the Gag polyprotein precursor that wraps the viral RNA. NC bears intrinsically disordered prion-like domains (PrLDs) and 2 conserved Cys3His zinc-finger motifs. In vitro experiments showed that HIV-1 NC protein condensed into spherical assemblies in the presence of crowding agents or cell homogenates. NC condensates exhibited properties of LLPS droplets, including shape, fluidity, rapid internal diffusion, and the ability to fuse and coalesce, and required an intact Cys3His motif [99]. Treatment with Zn^2+^ ejectors caused nuclear relocalization of NC and viral RNA and inhibition of virus release. Moreover, a common Zn2+-dependent LLPS-based mechanism of retroviral assembly has been suggested that would also impact on the ability of HIV-1 to regulate the assembly and disassembly of SGs during infection [99].

### DNA viruses

Herpes simplex virus 1 (HSV-1) was the first dsDNA virus hypothesized to perform replication in the context of LLPS, based on the fact that HSV-1 replication compartments, which recruit cellular RNA-Polymerase II, are spherical and fuse upon contact, and also on the fact that many HSV-1 proteins are predicted to be highly disordered [100–102]. However, a quantitative analysis suggested that the diffusion kinetics of these VRCs compared to the surrounding nucleoplasm would not be consistent with liquid-like properties [103]. Nevertheless, additional emerging evidence appears to support the involvement of proteins from HSV-1 and other members of the Herpesviridae family in the formation of BMCs. These include the HSV-1 transcription factor (TF) ICP4 [102] and HSV-1 UL11, the smallest conserved tegument protein among herpesviruses [104]. HSV-1 UL11 is an IDP, binds RNA, and undergoes LLPS in vitro, strongly suggesting that LLPS could be implicated in the assembly of the viral tegument layer located between the nucleocapsid and the lipid envelope [104]. The Epstein–Barr virus proteins EBNA2 and EBNALP, with roles in viral and cellular gene transcription, have been shown to mediate the formation of liquid-like condensates at superenhancer sites of cellular genes through their IDRs [105]. Another interesting example is the viral latency-associated nuclear antigen (LANA) from Kaposi’s sarcoma-associated herpesvirus, which associates with the viral genome to form dynamic LANA-nuclear bodies implicated in episome maintenance, through a mechanism partially mediated by LLPS [106].

Human papillomavirus (HPV) infection leads to the formation of the so-called replication foci or HPV E1/E2 foci, which contain the viral helicase E1 and the E2 master regulator and recruit DDR proteins [107]. HPV16 E2 can associate into ionic strength-dependent and readily reversible insoluble oligomers with both the E7 oncoprotein and with DNA [108,109]. While emerging evidence supports that compartmentalization of superenhancers is regulated by phase separation [110,111], formation of superenhancer-like elements has been postulated as a novel mechanism of HPV-16 integration [112], highlighting the potential role of LLPS in viral oncogenesis.

### Nucleoprotein and RNA-driven LLPS in SARS-CoV-2

The SARS-CoV-2 nucleocapsid (NCoV2) protein encapsulates and packages the approximately 30 kb viral RNA genome into the 80 to 100 nm membrane-enveloped virion and regulates viral gene transcription [113]. It has globular RNA binding and dimerization domains, flanked by 2 IDRs and a linker between them. Its modularity, multivalence, flexibility, RNA binding capacity, and IDRs point NCoV2 as a candidate for LLPS. Indeed, several groups showed that it undergoes cooperative LLPS upon binding to RNA [114–119]. Heterotypic LLPS occurs at an optimal RNA length and concentration, above which the process is inhibited [119,118], a phenomenon known as reentrant phase separation, related to the ratio of binding sites [120]. There appears to be some sequence preferences for binding but no evident sequence specificity for LLPS [115,116,118,119], as expected for a protein that binds throughout the entire genome. Interestingly, structural features of certain regions of the genomic RNA drive condensation while other regions dissolve it [117]. Moreover, high density condensates of N-RNA in cells recruit the RNA-dependent RNA polymerase, supporting the role of LLPS-based viral condensation in SARS-CoV-2 transcription and replication [115]. The membrane protein (M) promotes heterotypic LLPS with N, and 3-component mixtures of N, M, and RNA form condensates with mutually exclusive compartments containing N-M or N-RNA. Structures described as annular were observed, with the M protein coating the outer layer of the N-RNA droplets [119].

Phosphorylation at the central IDR of NCoV2 is required for transcription at the replication transcription complex [121]. While unphosphorylated N forms gel-like structures, phosphorylation leads to liquid-like droplets, showing how the material properties of the condensate can be modulated by PTM [114,119]. Thus, unmodified N leads to a structured oligomer more competent for nucleocapsid assembly, whereas the liquid-like condensate formed by the phosphorylated protein leads to a condensate better suited for viral RNA synthesis activity [119]. Another possible layer of modulation arises from the biphasic triggering/dissolving effect of ATP on N-RNA LLPS, where ATP binds at an RNA site with affinities in the range of cellular concentrations of the nucleotide [122], suggesting a role for ATP in the uncoating, localization, and packing of the RNA genome [122].

SGs are at the crossroads between the viral infection and host factors and are modulated by viruses to maximize replication efficiency [123]. NCoV2 was found to phase separate in vitro with human RNA binding proteins prone to LLPS (TDP-43, FUS, hnRNPA2), all associated with SG formation, suggesting a possible mechanism to co-opt host proteins [116]. N was also reported to be associated with SGs within cells [115,119] and was shown to interact with Ras-GTPase-activating protein SH3-domain-binding protein (G3BP) and disrupt SG assembly through its IDR1 [124,119]. Further, N blocks the interaction of G3BP1 with SG-related proteins, and the domains of N important for phase separation with G3BP and SG disassembly are required for viral production [124]. N-RNA-driven LLPS was also shown to recruit TK1 and IKK complex, both key kinases in the NF-kB signaling pathway, producing a NF-κB hyperactivation, proposed as a possible route to dysfunctional inflammatory response [125]. Despite most of the work on SARS CoV2 condensation has been obtained by in vitro experiments with recombinant and pure components, N condensation through LLPS was suggested to take place during infection, and a natural mutation (R203K/G204R) resulting in a nucleotide polymorphism in 37% of 100,000 genome sequences analyzed was associated with higher propensity of N to undergo LLPS and a more pronounced effect on interferon inhibition [126]. Finally, gallocatechin gallate (GCG), a polyphenol obtained from green tea, and which interferes with viral N-RNA complexes of other viruses, was found to inhibit SARS-CoV-2 replication likely through impairment of LLPS of N [126].

## Biochemical advantages of LLPS for viral factory formation, function, and fate

Viruses must replicate their genomes, transcribe their genes into mRNA using own or host enzymes, and rely on the cellular machinery for translation of viral proteins. The condensation of viral molecules into dynamic structures seems to be nature’s answer for these processes to be separated spatiotemporally. The most widely described VF assemblies to date are those of nsNSVs. We can picture how this might operate in the light of how their formation, function, and fate benefit from the chemistry behind LLPS. Fundamental features are at least in part shared even with viruses displaying different genome architecture and life cycles. We hypothesize a plausible set of sequential events from a biochemical perspective.

**Fig 2:**
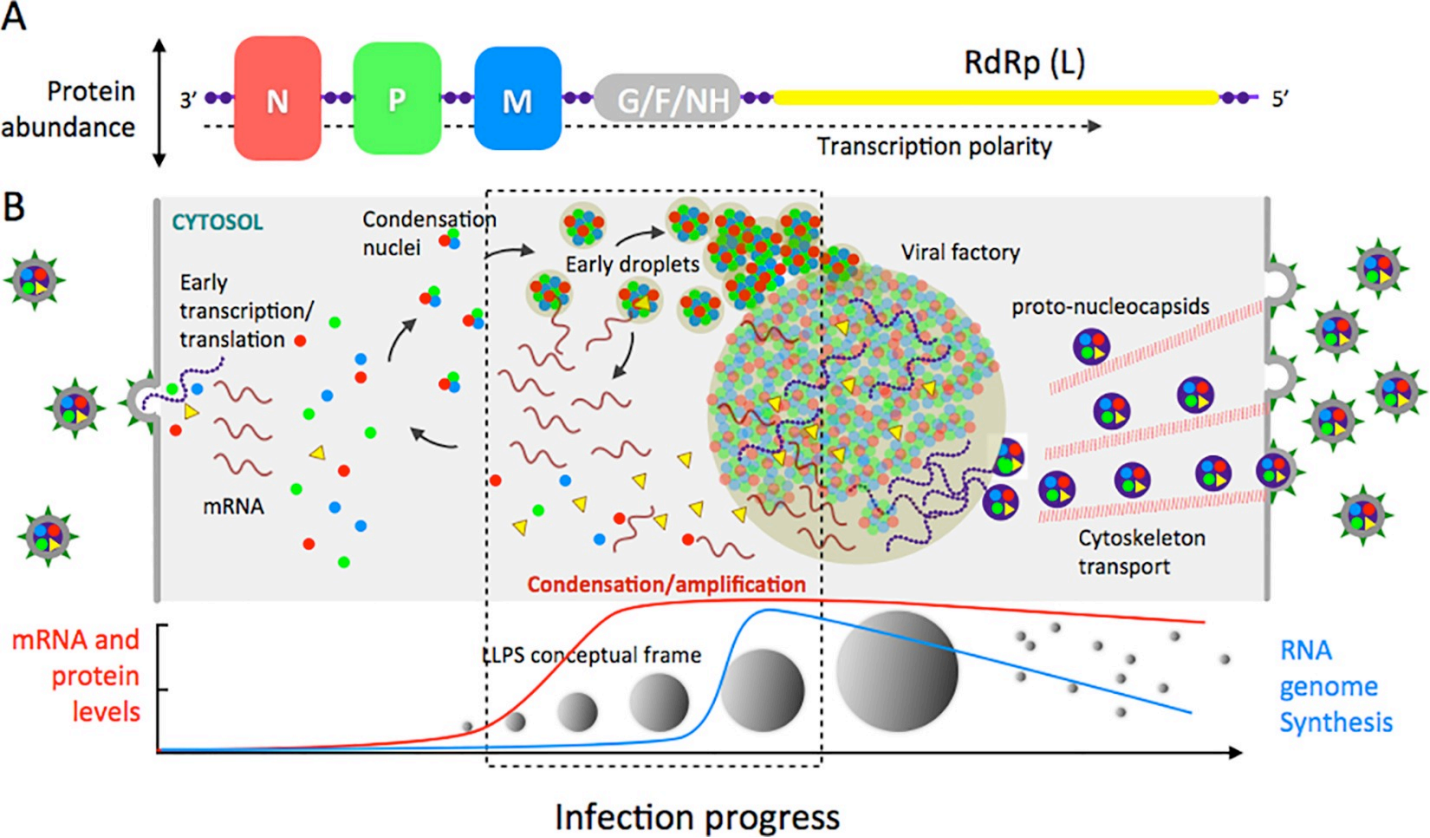
A general integrative model for condensation of VFs based on NSVs. **(A)** Simplified depiction of nsNSV genome structure. The viral transcription mechanism ensures a gradient of protein expression, with higher abundance of N, encoded at the 3′ region, and lower abundance of the large polymerase, invariably encoded at the 5′ end of nsNSV genomes. **(B)** Model depicting the formation of cytosolic VFs along viral infection. The schema stresses the amplification effect on RNA and protein synthesis caused by the self-primed formation of condensates (see main text). Toward the end of the infectious cycle, the immature nucleocapsids (proto-nucleocapsids) protrude from the VF and are transported along the cytoskeleton network to the assembly site [73]. The principle behind LLPS indicate that as the concentration of components increase, a partition into a newer denser phase takes place, where the concentration within the droplets remain constant, and further increase in the concentration of the components in the surrounding milieu cause an increment of the size of the condensates. The condensation event causes an abrupt increase of the effective local concentration of RNA and proteins, the drivers of the LLPS, which results in a substantial amplification of transcription and genome replication in the VF. LLPS, liquid–liquid phase separation; nsNSV, nonsegmented NSV; NSV, negative-stranded virus; RdRp, RNA-dependent RNA polymerase; VF, viral factory.

Upon entry, nsNSVs use the structural proteins carried within the infective virion for initial transcription. As protein levels increase and genome replication progresses, accumulated protein and RNA scaffolds nucleate and condense, recruiting viral and most likely several cellular clients. Additionally, the flexibility of the incoming vRNP and associated proteins could contribute to nucleate the LLPS process. A basic principle of LLPS is that the concentration of components within a condensate is constant, and increasing the concentration of components in the surrounding dilute phase leads to an increase in size of the condensate (Fig 1B). Further, a consequence of the transcription polarity of nsNSVs and their highly conserved gene arrangement is that those proteins encoded at the 3′ end of the genome will be produced first and at higher levels, with the late product invariably being the RNA-dependent RNA polymerase L (Fig 2A). While initial amounts of the vRdRp drive initial transcription and replication at minimum levels in the dilute cytosolic phase, the newly translated polymerase would enter at a later stage as client to the growing viral condensates.

As the condensates grow in size at the expense of the accumulation of components, transcription and replication rates would increase at least 1 order of magnitude, as a consequence of the concentration/condensation process [127] (Fig 2B). Such a mass action phenomenon largely increases the effective local concentration of both the polymerase, its nucleocapsid template and the NTP substrates and cofactors, maximizing RNA synthesis, capping, and methylation, resulting in transcription amplification [128]. This idea is supported by changes from linear to exponential increase of RNA synthesis in Paramyxoviruses [81,129,130]. Another possible and not mutually exclusive explanation is that de novo synthesis of vRdRp from the incoming template correlates with the change from a linear to an exponential vRNA accumulation [130]. The enlargement in RNA length is equivalent to an increment in concentration as multiple binding sites become available, added to the increase in RNA transcript molecules per se, both acting as LLPS scaffolds that further rise the size of the VF. At a certain level of transcript concentration and possibly through mechanism involving compositional/stoichiometric control, mature transcripts exit to the cytosol for further translation and protein accumulation into the condensates, generating a self-priming amplification cycle that maximize growth of the VFs to structures of up to 20 μm^2^ hosting a large amount of encapsidated genomes [78]. In addition to a size-limiting mechanism, the number of VFs is also limited to a few per cell [78]. At this point, capsid/genome packages of the approximate size of the virion are ejected from the factories and transported via cytoskeleton to the membrane, with the participation of the matrix (M) protein [131,132], to bridge and interact with the intracellular domains of the viral membrane glycoproteins, which were synthesized in the ER, before exiting the cell [133,132]. In the case of nsNSVs, packages ejected from the VFs include the N-encapsidated genome and the structural proteins that will be part of the virion (L, polymerase cofactor/s and M protein) (referred to as “proto-nucleocapsids” in Fig 2B). Besides the concentration of the macromolecules, compositional control, confinement, and environmental factors, PTMs should also be considered as an additional key layer of control over formation and disassembly of the viral condensates, particularly if modifying host enzymes are likely present in the condensate [134].

**Fig 3:**
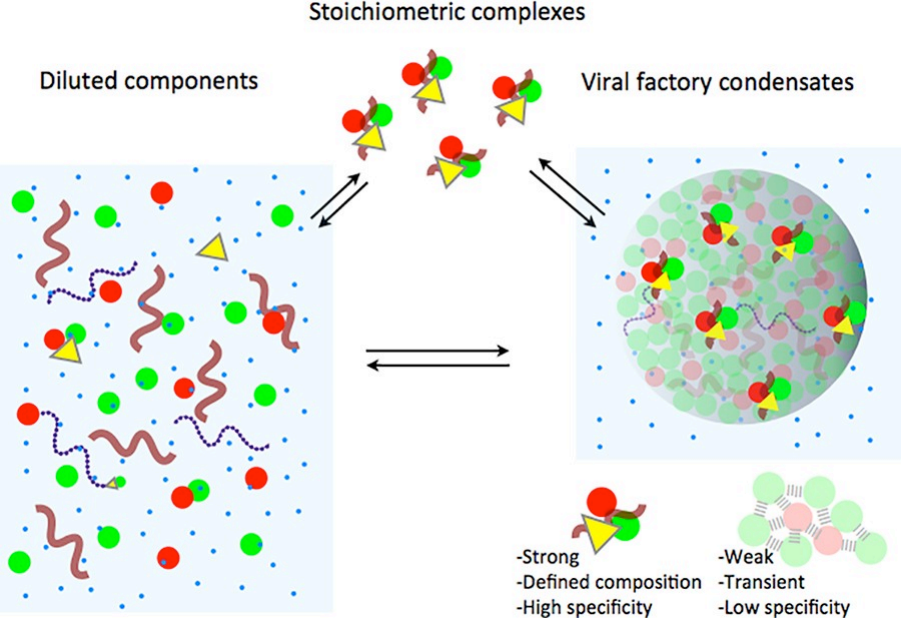
Nature of interactions in a viral condensate. Complexes of different molecularity between the replication/transcription machinery exist in solution below their respective association constant. Above that level, defined stoichiometric complexes are formed, which are also present within the dense phase, coexisting with excess free components, particularly those acting as scaffold or drivers for LLPS. These complexes are held together by strong and specific interaction. Conversely, the interactions holding the condensate are weak and transient, required for a modulated assembly/disassembly, and display low specificity. These low affinity interactions take place only in the condensate, where concentrations can be a few orders of magnitude higher than those in the diluted cytosolic phase. LLPS, liquid–liquid phase separation.

It should be noted that the whole process requires that the interactions within the VF are weak, dynamic, and reversible, all properties intrinsic to LLPS condensates. Some of the components of replication complexes form stoichiometrically defined species in solution based on specific interactions, in equilibrium with the free components (Fig 3). Interactions that hold the condensate together are, on the other hand, weak, transient, and of low specificity. Thus, the droplet or condensate itself is not an ordered complex, but rather a different liquid phase that includes stoichiometric complexes interacting weakly with the other components, including cellular factors (Fig 3).

## Conclusions and prospects

Virus genomes encode a higher proportion of disordered proteins than eukaryotic or bacterial genomes, which often interact with a number of cellular partners, implying multiple weak and nonspecific interactions. Viruses also require a number of cellular proteins for their perpetuation and the ability of viral proteins to establish multiple dynamic and weak interactions linked to the formation of phase-separated condensates provide a mechanism for as yet unidentified host proteins partitioning together with the viral machinery. The validation of the liquid nature of VFs has been already established, and the reconstitution of condensates from its essential components in vitro emerges as a fundamental tool to understand the underlying mechanisms that can be ultimately probed by reverse genetics and a battery of techniques being developed for investigating BMCs in cellulo. These systems not only provide insights into gene function across viral families but also contribute to the understanding of fundamental mechanisms of LLPS-based biomolecular condensation. They also provide valuable models for the understanding of BMC in cellular transcription and replication, manifested in changes in chromatin and the condensed nature of superenhancers. Finally, LLPS-associated pathological processes emerge as novel therapeutic targets, which definitely involves a new approach for antiviral discovery, complementing classical drug design and screening [135,136].
